# Predictive factors of response to liraglutide in patients with type 2 diabetes mellitus and metabolic syndrome

**DOI:** 10.3389/fendo.2024.1449558

**Published:** 2024-10-04

**Authors:** Jinfang Song, Na Li, Yongru Zhuang, Ya Chen, Chu Zhang, Jian Zhu

**Affiliations:** ^1^ Department of Pharmacy, Affiliated Hospital of Jiangnan University, Wuxi, China; ^2^ Jiangsu Key Laboratory of New Drug Research and Clinical Pharmacy, Xuzhou Medical University, Xuzhou, China; ^3^ Department of Pharmacy, Taizhou People’s Hospital, Taizhou, China; ^4^ Department of Endocrinology, Affiliated Hospital of Jiangnan University, Wuxi, China

**Keywords:** liraglutide, response, predictive factors, type 2 diabetes mellitus, metabolic syndrome

## Abstract

**Background:**

Although liraglutide has established advantages in treating patients with type 2 diabetes mellitus (T2DM) and metabolic syndrome (MS), there are still some patients with lower responsiveness to liraglutide. The objective of the study was to identify the predictors of response to liraglutide in patients with T2DM and MS.

**Methods:**

This retrospective cohort study included patients diagnosed with T2DM and MS who received liraglutide treatment as a part of their diabetes management for a minimum of six months. The participants were stratified into two groups: responders (HbA1c reduction≥1.0% and weight loss≥3%) and non-responders. The discrepancies in baseline data between the two groups were analyzed, containing comedications, test parameters, and basic profiles. The affecting factors of response to liraglutide by Logistic regression analysis were performed, and the predictive ability of the identified factors was evaluated by plotting a receiver operating characteristic (ROC) curve.

**Results:**

A total of 417 patients with T2DM and MS were examined and followed up according to the inclusion criteria, and 206 patients completed the follow-up; 105 (50.97%) were responders and 101 (49.03%) were non-responders to liraglutide. The binary logistic regression analysis identified baseline HbA1c, baseline BMI, and the duration of T2DM as significant predictors of glycemic and weight responses to liraglutide (*P <*0.05). The area under the curve of the ROC for the three predictors of liraglutide response after 6 months of treatment was 0.851 (95% confidence interval: 0.793 - 0.910).

**Conclusion:**

The baseline HbA1c, baseline BMI, and duration of T2DM were shown to be predictive factors of glycemic and weight improvements in patients with T2DM and MS treated with liraglutide, and had good predictive power.

## Background

Type 2 diabetes mellitus (T2DM) is a public health issue that seriously threatens the health of many people, and according to the International Diabetes Federation (IDF), there are 537 million people with diabetes mellitus worldwide, accompanied by the clinical dilemma that less than one-third of T2DM patients reach their glycemic control targets (1–3). Metabolic Syndrome (MS) is a common clinical syndrome characterized by the aggregation of various cardiovascular risk factors, such as obesity, impaired glucose and lipid metabolism, and hypertension, with a 70.0% to 80.0% probability of being combined with metabolic syndrome in patients with T2DM (4). In addition, achieving an aggressive weight loss of 5-10% for glycemic control and cardiovascular diseases (CVDs) risk reduction is considered beneficial for some patients (5). However, clinical evidence shows that only a limited kinds of antidiabetic medications are effective in reducing weight, and there are significant individual differences in efficacy (6). Therefore, individualized treatment for patients with T2DM and rational selection of antidiabetic drugs is a pressing issue in clinical treatment associated with glycemic control and improvement of weight.

Glucagon-like peptide-1 receptor agonists (GLP1RAs), such as liraglutide and beralutide, are important therapeutic agents for the treatment of T2DM and MS (7). Liraglutide is widely used in patients with T2DM and MS because of its proven benefits in lowering blood glucose, lipid metabolism and reducing body weight (8–10). Yet, owing to individual differences, liraglutide has poor efficacy in some patients as a hypoglycemic therapy, such as the LEAD-3 study, which found that 49% of patients did not achieve the control goal of hemoglobin A1c (HbA1c) <7% after taking liraglutide for 3 months (11–13). In addition, the weight loss effect of liraglutide varied significantly between patients with T2DM. It was found that, after 24 weeks of treatment with liraglutide, about 25% of the participants showed weight loss <3%, and some of them even presented weight gain (13–15).

It is essential to pinpoint these predictors of response to GLP-1 RA treatment in the clinic, as this helps physicians optimize treatment regimens for patients with T2DM and MS. Consequently, the research aimed to explore the factors related to responses to the treatment of liraglutide in patients with T2DM and MS and to assess their predictive ability.

## Methods

### Patients

The study began with a search for information and initial data collection with records of liraglutide consumption, based on type 2 diabetes and metabolic syndrome as the primary diagnoses. The retrospective and observational study continued six months and involved 417 patients with T2DM and MS who received liraglutide treatment and their responses to treatment were assessed. The participants were sourced from the electronic medical record system between January 2021 and June 2022 at the Affiliated Hospital of Jiangnan University and the Affiliated Hospital of Xuzhou Medical University. All the patients were assigned to liraglutide at a dose of 0.6 mg injected s.c. once per day and increased to 1.2 mg/day after 1 week for six months. Data were compiled from eligible patients: (1) Fulfilled the diagnostic standards for T2DM and MS defined by the American Diabetes Association guideline (16). (2) Patients aged 18-75 without gender restrictions. (3) Patients with comprehensive medical records. (4) Received liraglutide treatment for a minimum of six months prior to data collection. Exclusion criteria: (1) Diagnosed as other subtypes of diabetes; (2) Combined with serious complications of diabetes; (3) Patients with severe organ dysfunctions or malignant tumors; (4) Patients previously treated with a GLP-1R analog; (5) Medical data recorded incompletely. Therefore, only 206 participants were involved in the final analyses. Ethics application was approved by our institutional review board. This study was approved by the Ethics Committee of the Affiliated Hospital of Jiangnan University (NO. LS2021091).

### Measurement of the anthropometric and biochemical parameters

The clinical characteristics included age; sex; duration of T2DM; history of smoking; history of drinking; family history of diabetes; body mass index (BMI); waist-to-hip ratio (WHR). Plasma glucose and serum lipids, including total cholesterol (TC); triglyceride (TG); high-density lipoprotein cholesterol (HDL-c); low-density lipoprotein cholesterol (LDL-c); HbA1c; insulin level; and concomitant diabetic therapies before and after treatment. In addition, insulin resistance and beta-cell function were evaluated as mentioned formerly (17).

### Study cohorts

Participants were stratified into non-responders and responders based on their glycemic and weight response to liraglutide treatment. According to the National Institute for Health and Care Excellence (NICE) guidelines for using GLP-1 receptor agonists in the treatment of T2DM, glycemic response was defined as reduction in HbA1c of at least 1.0% and weight response was defined as a weight loss of at least 3% compared to baseline after six months of administration with GLP-1 receptor agonists (18). Therefore, responders were identified as individuals who achieved a reduction in HbA1c of at least 1.0% and a weight loss of at least 3% following six months of administration with liraglutide, conversely, non-responders were those who did not achieve this standard in this study.

### Statistical analysis

Statistical analyses were performed with SPSS software (version 16.0, SPSS Inc., USA). The data are presented as percentages or the mean ± standard deviation (SD), according to the condition. Differences in clinical indicators before and after treatment were compared using paired-samples t-test. Baseline characteristics between responders and non-responders were evaluated using the Chi-squared test or Fisher’s exact test for categorical variable and independent Student’s t test for continuous variable. Binary logistic regression analysis was applied to determine the independent predictors of response to liraglutide. The area under the curve (AUC) of the receiver operating characteristic (ROC) curve and the 95% CI were computed to assess the predictive efficacy of the predictors. A value of *P* < 0.05 was acknowledged statistically significant.

## Results

### Baseline characteristics of all the subjects

In accordance with the inclusion and exclusion standard, the final analysis included 206 patients (137 men and 69 women). Then, according to the explanation of responders and non-responders, the 206 patients were stratified into responders (105 patients) and non-responders (101 patients). The baseline characteristics of the individuals are summarized in **Table 1**. In contrast to the responder group, the non-responder group exhibited lower mean values for HbA1c (*P* < 0.01), weight (*P* < 0.001), and BMI (*P* < 0.01), but a longer duration of T2DM (*P* < 0.001). No significant differences were observed between the two groups regarding the other baseline characteristics.

**Table 1:** Comparison of baseline characteristics between responders (n=105) and non-responders (n=101).

Baseline characteristics	Group		*P* value
	Responders	Non-responders	
N(men/women)	105 (69/36)	101 (68/33)	0.806
Age (years)	45.08 ± 11.87	47.99 ± 12.09	0.083
Duration of T2DM (years)	5.32 ± 2.21	7.99 ± 4.68	0.000
Smoking history (%)	35 (33.33)	31 (30.69)	0.685
Drinking history (%)	29 (27.62)	32 (31.68)	0.523
Weight (kg)	83.21 ± 12.02	76.65 ± 11.48	0.000
BMI (kg/m^2^)	28.58 ± 4.13	26.89 ± 3.42	0.002
FPG (mmol/L)	10.41 ± 2.35	9.90 ± 2.71	0.150
PPG (mmol/L)	16.08 ± 3.87	14.70 ± 4.51	0.019
HbA1c (%)	9.87 ± 1.56	9.24 ± 1.51	0.003
TC (mmol/L)	5.26 ± 1.52	5.07 ± 1.23	0.326
TG (mmol/L)	2.72 ± 2.34	2.27 ± 1.70	0.117
HDL-c (mmol/L)	1.27 ± 0.45	1.22 ± 0.36	0.381
LDL-c (mmol/L)	3.04 ± 1.01	2.98 ± 0.98	0.666
Family history of diabetes (%)	20.95	21.78	0.884
Liraglutide only (%)	22 (20.95)	23 (22.77)	0.752
Liraglutide + OHAs (%)	43 (40.95)	33 (32.67)	0.218
Liraglutide + Insulin (%)	16 (15.24)	15 (14.85)	0.938
Liraglutide + OHAs +Insulin (%)	24 (22.86)	30 (29.70)	0.264

T2DM, type 2 diabetes mellitus; BMI, body mass index; FPG, fasting plasma glucose; PPG, postprandial plasma glucose; HbA_1c_, hemoglobin A_1c_; TC, total cholesterol; TG, triglyceride; HDL-c, high-density lipoprotein-cholesterol; and LDL-c, low-density lipoprotein-cholesterol; OHAs, oral antihyperglycaemic agents.

### Alterations in clinical parameters at each time point following liraglutide therapy

**Figure 1:**
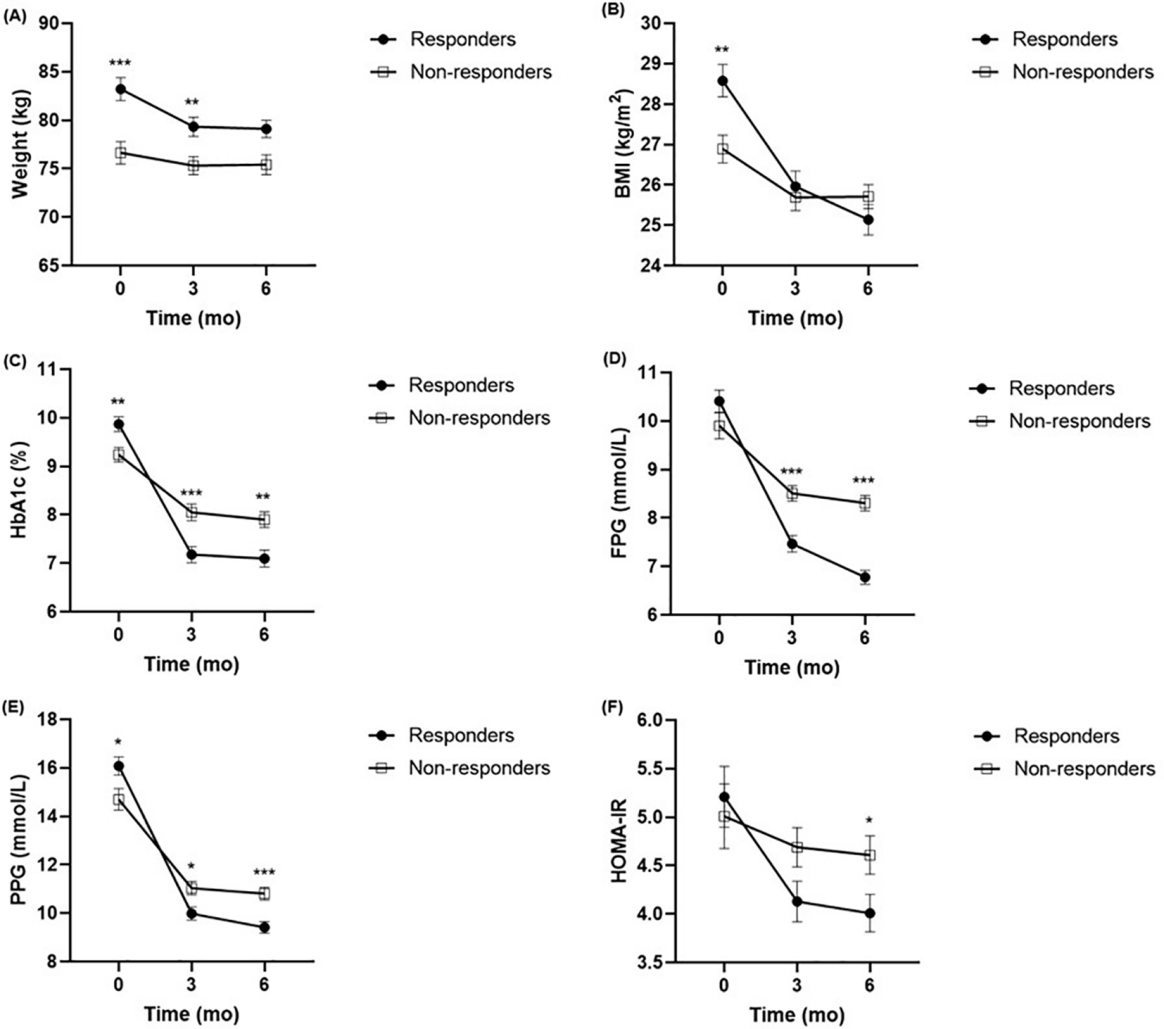
Comparison of body weight **(A)**, BMI **(B)**, HbA1c **(C)**, FPG **(D)**, PPG **(E)**, HOMA-IR **(F)** between responders (n = 105) and non-responders (n = 101) at baseline, 3 months and 6 months. ^*^ *P* <.005, ^**^ *P* < 0.01, ^***^ *P* < 0.001 compared with non-responders. BMI, body mass index; HbA1c, haemoglobin A1c; FPG, fasting plasma glucose; PPG, postprandial plasma glucose; HOMA-IR, homeostasis model assessment for insulin resistance.

The alterations in the clinical parameters from baseline to 6 months after liraglutide treatment are shown in **Supplementary Tables S1**, **S2**. The results showed significant improvements in glucolipid metabolism in each group after treatment with liraglutide, as evidenced by improvements in body weight, BMI, FPG, PPG, HbA1c, PINS, HOMA-IR, HOMA-B, TC, and LDL-c. Furthermore, comparing the clinical parameters between the two groups at baseline, 3 months, and 6 months, respectively (**Figure 1**). In contrast to the non-responders, body weight and BMI levels were higher in the responders at baseline, but the difference had disappeared at 6 months (**Figures 1A, B**). In contrast to the non-responders, HbA1c and PPG levels were higher in the responders at baseline, but lower at 3 months, and 6 months (**Figures 1C, E**). The FPG and HOMA-IR were not significantly different between the two groups at baseline, but they were lower in responders than in non-responders (**Figures 1D, F**). There were no significant differences in FINS, PINS, HOMA-B, TG, TC, HDL-c, and LDL-c detected between the responders and non-responders throughout the observation period.

### The function of baseline characteristics in predicting response to liraglutide treatment

**Figure 2:**
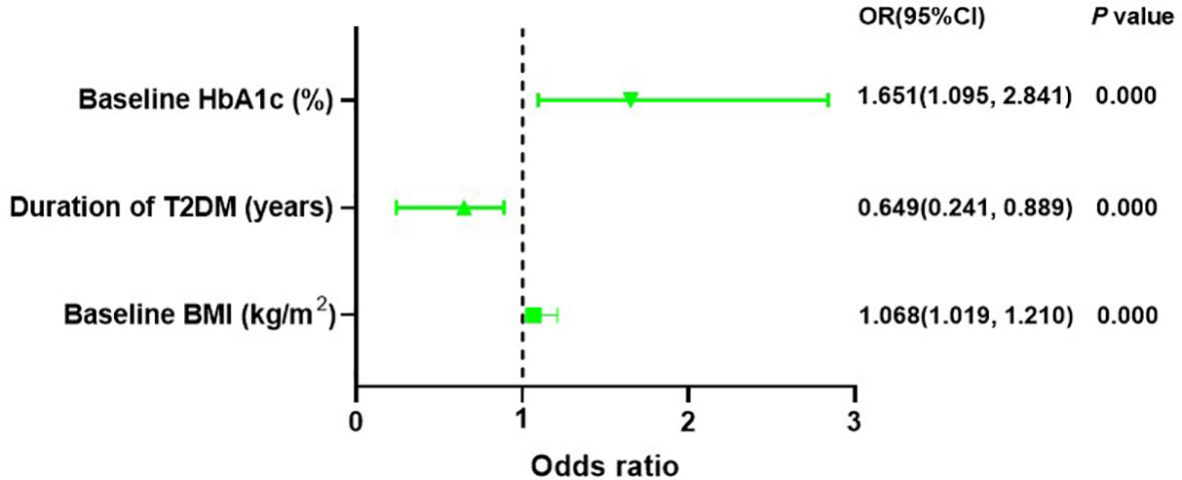
Binary logistic regression recognized baseline HbA1c, baseline BMI and duration of T2DM can forecast response to liraglutide treatment. HbA1c, haemoglobin A1c; T2DM, type 2 diabetes mellitus; BMI, body mass index.

A binary logistic regression analysis was conducted to assess the impact of baseline characteristics and clinical indicators on response to liraglutide. In addition, patients with higher baseline BMI (OR = 1.068, CI: 1.019-1.210, *P* = 0.005) and baseline HbA1c (OR = 1.651, CI: 1.095-2.841, *P* = 0.000) or with a shorter duration of T2DM (OR=0.649, CI: 0.241-0.889, *P* = 0.000) were more possibly classified as responders to liraglutide treatment (**Supplementary Table S3**, **Figure 2**).

### ROC curves for the predictors of response to liraglutide

**Figure 3:**
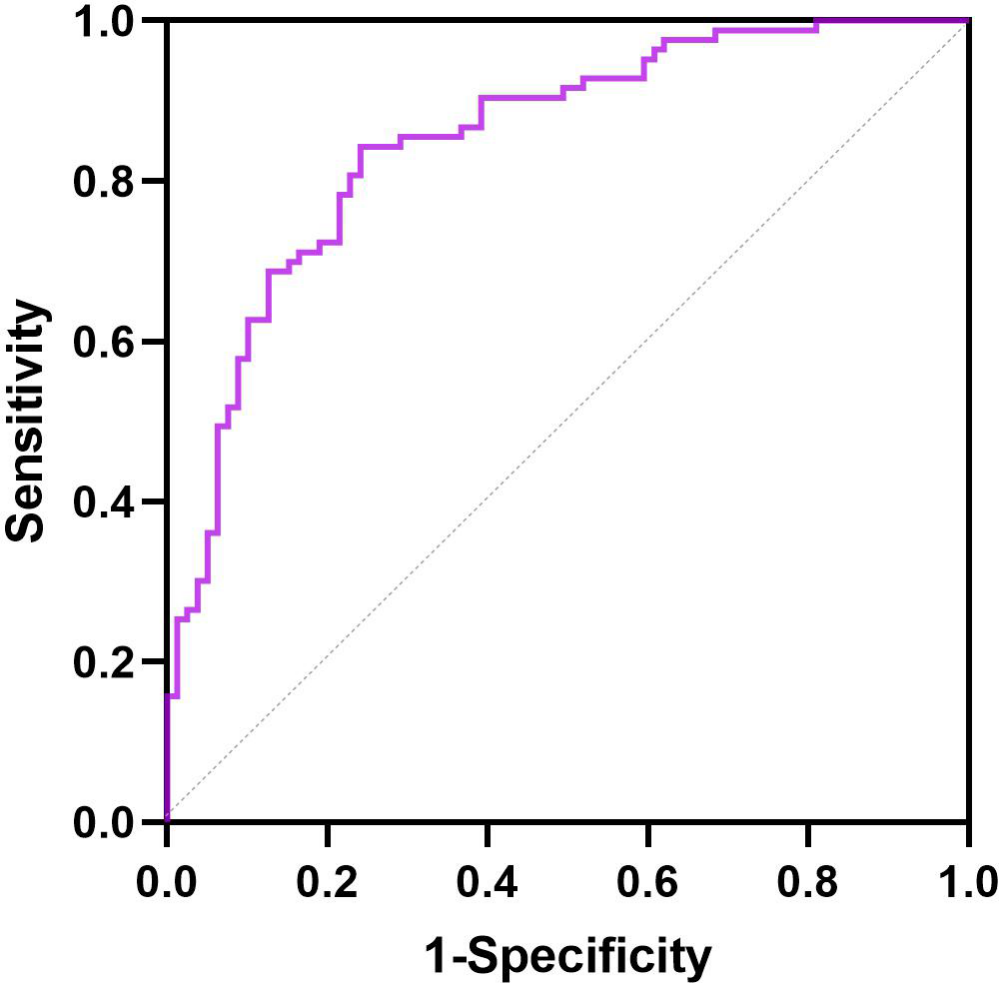
ROC curve for the predictive factors of response to liraglutide in patients with T2DM and MS.

The ROC curves of the response to liraglutide for baseline BMI, baseline HbA1c, and duration of T2DM are presented in **Figure 3**. The area under the ROC curve for the predictive factors of HbA1c reduction and weight loss within 6 months of liraglutide initiation was 0.851 (95% CI: 0.793–0.910). Moreover, the area under the ROC curve was conducted to identify the ability of the predictive factors of response to liraglutide treatment (**Supplementary Figure S1**). The areas under the ROC curves were 0.667 (95% CI: 0.585–0.749) for baseline BMI, 0.775 (95% CI: 0.705–0.846) for baseline HbA1c, and 0.728 (95% CI: 0.648–0.809) for duration of T2DM.

### Safety

Responders and non-responders have favorable tolerance to liraglutide. Of the 206 participants finally involved, 42 (18.45%) experienced tolerable adverse gastrointestinal events (GIAEs). The incidence of GIAEs was equilibrated between the two groups (*P* >0.05).

## Discussion

This study investigated possible predictive factors of glycemic and weight responses to liraglutide in patients with T2DM and MS. The results indicated that baseline HbA1c, baseline BMI, and duration of T2DM are significant predictors of response to liraglutide, and that patients satisfying the standards of higher baseline HbA1c, higher baseline PPG, and a shorter duration of T2DM are more effective on liraglutide treatment. Therefore, our findings will be of interest in guiding the individualized clinical application of liraglutide.

Sample size calculations were considered with HbA1c and BMI as study endpoints. According to the results of this study, patients in the responder group were more sensitive to liraglutide treatment than patients in the non-responder group. After 6 months of treatment, the responding group had an additional 1.43 per cent reduction in HbA1c and an extra 2.26 reduction in BMI. According to the bilateral α=0.05 and statistical power>80%, the maximum standard deviation (σ) of HbA1c value in this study was 1.56%, and the σ of BMI value was 4.13 for calculation. Calculate the sample size based on HbA1c: N=[(Zα/2+Zβ)^2^×{2×(σ)^2^}]/(μ_1_-μ_2_)^2^, then N=[(1.96 + 0.84)^2^×{2×(1.56)^2^}]/(1.43)^2 =^ 19; Similarly, based on BMI, the sample size is calculated as N=53. Considering that patients may have a dropout rate of 10%, there are 59 patients in the non response group and 62 patients in the non response group, which can provide sufficient statistical power. Based on the above analysis, the total sample size selected for this study is 206 patients, with 101 patients in the non response group and 105 patients in the response group, ensuring a statistical power of over 80%. Therefore, the conclusions drawn from the research based on the current sample size are reliable.

Accumulating evidence has reported that liraglutide improved not only glycemic control, but also weight loss (18–20). In setting the responsiveness grouping criteria, HbA1c decrease ≥1.0% and weight loss ≥3% were considered the criterion for response grouping according to the Guideline for the Prevention and Treatment of Type 2 Diabetes Mellitus in China (2020 edition) (19). However, according to the National Institute for Health and Care Excellence (NICE) guidelines on the use of GLP-1 receptor agonists, a glycemic response is defined as a >1.0% reduction in HbA1c from baseline at 6 months of treatment with GLP-1 receptor agonists (18). It was found in the LEAD-3 study that the optimal glucose-lowering efficacy was achieved with liraglutide alone at a dose of 1.8 mg for 3 months, with a decrease in HbA1c of about 1% from baseline levels (13). By contemplating the above standards together, responders were identified as individuals who achieved a reduction in HbA1c of at least 1.0% and a weight loss of at least 3% following six months of administration with liraglutide, conversely, non-responders were those who did not achieve this standard in this study. On this basis, the current study found that only 50.97% of patients with T2DM and MS who had been treated with liraglutide for 6 months met the criteria for the determination of responders. Previous studies have shown that approximately 35%-56.5 of patients are GLP-1RAs treatment non-responders based on HbA1c levels before and after GLP1RA treatment, which is consistent with our findings (11, 12). However, it should be noted that the requirements for meeting the standard in the responders group in this study were more critical, not only evaluating glycaemic control (reduction in HbA1c of at least 1.0%), but also meeting the restriction of weight loss of more than 3%, which would certainly increase the proportion of non-responders to a certain extent. In addition, it should be noted that the current study had a follow-up dose of liraglutide of 1.2 mg/day and did not include patients treated with 1.8 mg/day, which would also necessarily increase the proportion of non-responders due to the smaller dosage of the drug. There were significant individual differences in efficacy and adverse drug reactions when different patients were treated with an identical dose of GLP-1RAs (13, 21, 22). Such variations in treatment response may result from discrepancies in living environments, medication adherence, the genetic background of the patient, and pathophysiological condition. Differences in living environments, the genetic background of the patient, medication compliance, and pathophysiological status may contribute to such variations in treatment response. Therefore, it is necessary to explore and define the predictors of individual differences in response to liraglutide treatment in patients with T2DM and MS, which is important for the individualization of liraglutide administration.

The present study also indicated that baseline HbA1c, baseline BMI, and duration of T2DM were predictors of response following six months of liraglutide treatment. We initially identified predictive factors that could concomitantly predict response to liraglutide regarding glycemia and body weight in patients with T2DM and MS. Clinical studies have revealed that patients with a long duration of T2DM have poorer pancreatic islet β-cell function and weaker responses to drug action, which suggests poorer glucose-lowering efficacy in patients with a long duration of T2DM treated with liraglutide and is consistent with our findings (21). According to previous studies, body weight was considered to be an important factor affecting the response to liraglutide based on the theory that liraglutide, as a GLP-1 agonist, has a strong effect on reducing glycemic and body weight, and patients with an increased body weight tend to have a higher glycemic level, resulting in more pronounced glycemic control efficacy after liraglutide treatment (22, 23). The predicative role of baseline glycemic control in predicting the efficacy response to liraglutide has been explored in multiple clinical studies but the results have not been conclusive (24, 25). One real world study in Italy suggested that better response to liraglutide glycemic therapy in some patients was not associated with baseline HbA1c levels (24). However, a study of the factors influencing the therapeutic efficacy of exenatide conducted in a Chinese population found that the baseline HbAlc of patients was an independent predictor of response to exenatide (25). While the present study observed a predictive effect of baseline HbA1c, and baseline BMI on responsiveness to liraglutide, but the previous studies suggest that different outcomes may be obtained in different populations and under different treatment regimens, therefore, more studies are needed to demonstrate the effect.

Regarding the discriminative abilities of predictive capability, our ROC curve analysis indicated that the AUC was effective in predicting HbA1c and weight reduction within 6 months following liraglutide treatment. The AUC values for the duration of T2DM, baseline BMI, and baseline HbA1c were 0.728 (95%CI 0.648-0.809), 0.667 (95%CI 0.585-0.749), and 0.775 (95%CI 0.05-0.846), respectively. Despite some researches have explored the predictive factors of response to liraglutide, relatively few have sought to develop ROC curves to evaluated the predictive capability of these predictors after they were recognized through binary logistic regression analysis. As for adverse reactions, 42 (18.45%) cases with tolerable GIAEs were detected in this study, which is consistent with the reports of studies conducted based on Asian populations, such as Chinese and Japanese populations, mostly occurring within 1-2 weeks of the first dose, mostly tolerable, and gradually decreasing as the treatment duration increased (12, 13). Therefore, the study supplies valuable insights into the practical apply of liraglutide. Depending on existing research advancements, the impact of clinical factors on the HbA1c response to liraglutide should be thoroughly regarded in clinical practice. Conducting more large-scale studies would be helpful for structuring individualized dosing models for liraglutide, enabling the development of accurate medical tools for patients.

There are several unresolved problems that demand further investigation in this study. Firstly, the results related to baseline weight, baseline HbA1c, baseline BMI, and duration of T2DM forecasting the response to liraglutide require repetition because they are resulted from significant variations in the responses of a relatively limited participant pool. Secondly, the retrospective analysis has some drawbacks in that data related to some influencing factors are often absent in electronic medical record systems, for example, lifestyle changes during follow-up, so we could not rule out the effect of other unrecorded influencing factors on weight loss and glycemic reduction. Finally, genetic factors may play a crucial role in influencing drug efficacy; however, no pharmacogenomic research of liraglutide has been performed. There is a necessity to further enhance the standard for evaluating the hypoglycemic efficacy of liraglutide and to improve the rational application of clinical drug.

In summary, this study determined predictive factors of response to liraglutide in patients with T2DM and MS by conducting a retrospective analysis, providing a foundation for guiding rational clinical application. The baseline HbA1c, baseline BMI, and duration of T2DM could be separately applied to forecast the response to liraglutide in patients with T2DM and MS. Significant predictors of the response to liraglutide can contribute to establish the groundwork for customizing a more accurate treatment for T2DM and MS.

## Data Availability

The original contributions presented in the study are included in the article/**Supplementary Material**. Further inquiries can be directed to the corresponding authors.

## Supplementary material

Supplementary Figure 1 ROC curves for the baseline weight, baseline BMI, duration of T2DM, and baseline HbA1c to show the efficiencies of the predictive factors of response to liraglutide treatment. HbA1c, haemoglobin A1c; T2DM, type 2 diabetes mellitus; BMI, body mass index.
