# Supplementary figures and images for: Predictive factors of response to liraglutide in patients with type 2 diabetes mellitus and metabolic syndrome
Source: Front Endocrinol (Lausanne). 2024 Oct 4;15:1449558. doi: 10.3389/fendo.2024.1449558 (PMC11486649; doi:10.3389/fendo.2024.1449558)

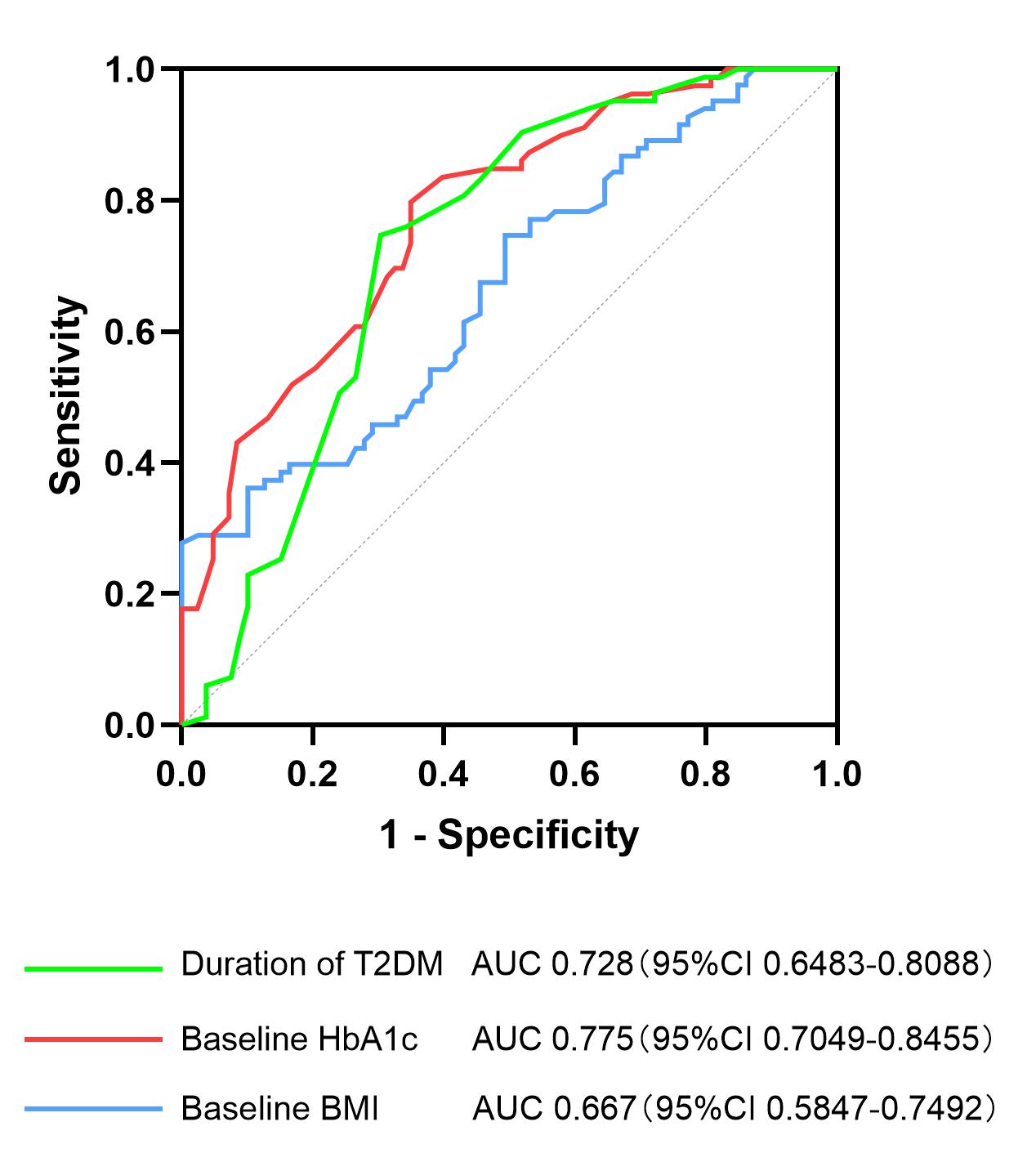

Supplement: Supplementary Figure 1 — ROC curves for the baseline weight, baseline BMI, duration of T2DM, and baseline HbA1c to show the efficiencies of the predictive factors of response to liraglutide treatment. HbA1c, haemoglobin A1c; T2DM, type 2 diabetes mellitus; BMI, body mass index. [file Image1.jpg]
