# Supplementary material for: Predictive factors of response to liraglutide in patients with type 2 diabetes mellitus and metabolic syndrome
Source: Front Endocrinol (Lausanne). 2024 Oct 4;15:1449558. doi: 10.3389/fendo.2024.1449558 (PMC11486649; doi:10.3389/fendo.2024.1449558)
Supplement: Supplementary file 2 [file Table1.docx]

**Table S1** Clinical characteristics of patients with T2DM and MS at baseline, 3 months and 6 months after liraglutide treatment within responders (n = 105)

| **Parameters** | **Follow up points** | | | **Overall**  ***P* value** | **Adjusted *P* value** | | |
| --- | --- | --- | --- | --- | --- | --- | --- |
|  | **Baseline** | **3 months** | **6 months** |  | **Baseline to 3 months** | **3 to 6 months** | **Baseline to 6 months** |
| Weight (kg) | 83.21±12.02 | 79.34±10.11 | 79.11±9.21 | 0.000 | 0.000 | 0.853 | 0.000 |
| BMI (kg/m^2^) | 28.58±4.13 | 25.96±3.91 | 25.14±3.87 | 0.000 | 0.000 | 0.000 | 0.000 |
| FPG (mmol/L) | 10.41±2.35 | 7.47±1.72 | 6.78±1.48 | 0.000 | 0.000 | 0.000 | 0.000 |
| PPG (mmol/L) | 16.08±3.87 | 9.99±2.76 | 9.42±2.41 | 0.000 | 0.000 | 0.000 | 0.000 |
| HbA1c (%) | 9.87±1.56 | 7.18±1.38 | 7.10±1.17 | 0.000 | 0.000 | 0.000 | 0.000 |
| FINS (mU/L) | 11.44±6.91 | 12.55±7.25 | 13.96±7.75 | 0.116 | 0.000 | 0.000 | 0.000 |
| PINS (mU/L) | 32.46±19.58 | 41.39±25.76 | 48.27±31.04 | 0.000 | 0.000 | 0.000 | 0.000 |
| HOMA-IR | 5.21±3.21 | 4.13±2.16 | 4.01±1.98 | 0.000 | 0.000 | 1.000 | 0.000 |
| HOMA-B | 38.75±30.73 | 76.41±55.65 | 107.79±98.62 | 0.000 | 0.000 | 0.000 | 0.000 |
| TC (mmol/L) | 5.26±1.52 | 4.68±1.06 | 4.51±1.13 | 0.000 | 0.000 | 0.003 | 0.000 |
| TG (mmol/L) | 2.72±2.34 | 2.01±1.48 | 1.97±1.41 | 0.000 | 0.001 | 1.000 | 0.000 |
| HDL-c (mmol/L) | 1.27±0.45 | 1.22±0.33 | 1.20±0.32 | 0.130 | 0.671 | 0.306 | 0.327 |
| LDL-c (mmol/L) | 3.04±1.01 | 2.83±1.01 | 2.72±1.04 | 0.011 | 0.170 | 0.090 | 0.020 |

BMI = body mass index; FPG = fasting plasma glucose; PPG = postprandial plasma glucose; HbA_1c_ = hemoglobin A_1c_; FINS = fasting serum insulin; PINS = postprandial serum insulin; HOMA-IR = homeostasis model assessment for insulin resistance; HOMA-B = homeostasis model assessment for beta cell function; TC = total cholesterol; TG = triglyceride; HDL-c = high-density lipoprotein-cholesterol; and LDL-c = low-density lipoprotein-cholesterol.

**Table S2** Clinical characteristics of patients with T2DM and MS at baseline, 3 months and 6 months after liraglutide treatment within non-responders (n = 101)

| **Parameters** | **Follow up points** | | | **Overall**  ***P* value** | **Adjusted *P* value** | | |
| --- | --- | --- | --- | --- | --- | --- | --- |
|  | **Baseline** | **3 months** | **6 months** |  | **Baseline to 3 months** | **3 to 6 months** | **Baseline to 6 months** |
| Weight (kg) | 76.65±11.48 | 75.32±9.46 | 75.42±10.32 | 0.000 | 0.000 | 0.958 | 0.000 |
| BMI (kg/m^2^) | 26.89±3.42 | 25.69±3.24 | 25.71±3.01 | 0.000 | 0.000 | 1.000 | 0.000 |
| FPG (mmol/L) | 9.90±2.71 | 8.51±1.60 | 8.31±1.58 | 0.000 | 0.000 | 0.993 | 0.000 |
| PPG (mmol/L) | 14.70±4.51 | 11.04±2.81 | 10.81±2.63 | 0.000 | 0.000 | 0.157 | 0.000 |
| HbA1c (%) | 9.24±1.51 | 8.05±0.96 | 7.90±1.01 | 0.000 | 0.000 | 0.041 | 0.000 |
| FINS (mU/L) | 11.58±6.84 | 12.78±7.39 | 13.72±6.98 | 0.002 | 0.064 | 0.030 | 0.002 |
| PINS (mU/L) | 36.19±23.94 | 42.12±26.17 | 46.11±29.59 | 0.001 | 0.007 | 0.008 | 0.000 |
| HOMA-IR | 5.01±3.34 | 4.69±2.04 | 4.61±2.00 | 0.003 | 0.004 | 0.446 | 0.139 |
| HOMA-B | 44.00±34.74 | 76.10±62.49 | 95.68±146.31 | 0.000 | 0.000 | 0.178 | 0.002 |
| TC (mmol/L) | 5.07±1.23 | 4.69±1.01 | 4.52±0.97 | 0.000 | 0.001 | 0.007 | 0.000 |
| TG (mmol/L) | 2.27±1.70 | 1.99±1.53 | 2.03±1.65 | 0.206 | 0.225 | 1.000 | 0.466 |
| HDL-c (mmol/L) | 1.22±0.36 | 1.24±0.40 | 1.20±0.37 | 0.030 | 1.000 | 0.025 | 1.000 |
| LDL-c (mmol/L) | 2.98±0.98 | 2.84±0.92 | 2.68±0.91 | 0.000 | 0.328 | 0.045 | 0.003 |

BMI = body mass index; FPG = fasting plasma glucose; PPG = postprandial plasma glucose; HbA_1c_ = hemoglobin A_1c_; FINS = fasting serum insulin; PINS = postprandial serum insulin; HOMA-IR = homeostasis model assessment for insulin resistance; HOMA-B = homeostasis model assessment for beta cell function; TC = total cholesterol; TG = triglyceride; HDL-c = high-density lipoprotein-cholesterol; and LDL-c = low-density lipoprotein-cholesterol.

**Table S3** Binary logistic regression analysis variables that can predict response to liraglutide treatment

| **Variables** | **Odds Ratio** | **95% CI** | ***P* value** |
| --- | --- | --- | --- |
| Age (years) | 1.028 | (9.630, 1.103) | 0.376 |
| Sex | 0.947 | (0.916, 1.363) | 0.765 |
| Smoking history (%) | 1.099 | (0.677, 1.465) | 0.835 |
| Drinking history (%) | 1.145 | (0.331, 3.962) | 0.830 |
| Family history of diabetes (%) | 1.538 | (0.931, 2.362) | 0.264 |
| Duration of T2DM (years) | 0.649 | (0.241, 0.889) | 0.000 |
| Baseline BMI (kg/m^2^) | 1.068 | (1.019, 1.210) | 0.005 |
| Baseline FPG (mmol/L) | 0.970 | (0.541, 1.535) | 0.708 |
| Baseline PPG (mmol/L) | 1.231 | (0.825, 1.566) | 0.426 |
| Baseline HbA1c (%) | 1.651 | (1.095, 2.841) | 0.000 |
| Baseline FINS (mU/L) | 1.458 | (0.868, 2.120) | 0.141 |
| Baseline PINS (mU/L) | 1.010 | (0.949, 1.102) | 0.934 |
| Baseline TC (mmol/L) | 0.932 | (0.494, 2.099) | 0.967 |
| Baseline TG (mmol/L) | 1.194 | (0.937, 1.612) | 0.375 |
| Baseline HDL-c (mmol/L) | 1.407 | (0.459, 2.623) | 0.677 |
| Baseline LDL-c (mmol/L) | 1.132 | (0.448, 2.149) | 0.816 |
| OHAs only | 1.061 | (0.245, 1.912) | 0.718 |
| Insulin only | 0.726 | (0.099, 2.031) | 0.816 |
| OHAs and Insulin | 0.629 | (0.201, 2.112) | 0.749 |

Abbreviations: OR = odds ratio; CI = confidence interval; T2DM = type 2 diabetes mellitus; BMI = body mass index; HbA1c = haemoglobin A1c; FPG = fasting plasma glucose; PPG = postprandial plasma glucose; FINS = fasting serum insulin; PINS = postprandial serum insulin; PPG = postprandial plasma glucose; TC = total cholesterol; TG = triglycerides; HDL-c = high-density lipoprotein cholesterol; LDL-c = low-density lipoprotein cholesterol; OHAs = oral antihyperglycaemic agents.
